# Corrosion Behavior of Ferritic 12Cr ODS and Martensitic X46Cr13 Steels in Nitric Acid and Sodium Chloride Solutions

**DOI:** 10.3390/ma17143466

**Published:** 2024-07-12

**Authors:** Krzysztof Nowik, Rafał Zybała, Bogna Sztorch, Zbigniew Oksiuta

**Affiliations:** 1 Institute of Mechanical Engineering, Faculty of Mechanical Engineering, Białystok University of Technology, Wiejska 45C, 15-351 Białystok, Poland; 2 Łukasiewicz Research Network—Institute of Microelectronics and Photonics, Al. Lotników 32/46, 02-668 Warsaw, Poland; rafal.zybala@pw.edu.pl; 3 Centre for Advanced Technologies, Adam Mickiewicz University in Poznań, Uniwersytetu Poznańskiego 10, 61-614 Poznań, Poland; bogna.sztorch@amu.edu.pl; 4 Institute of Biomedical Engineering, Faculty of Mechanical Engineering, Białystok University of Technology, Wiejska 45C, 15-351 Białystok, Poland

**Keywords:** oxide dispersion-strengthened (ODS) steel, ferritic steel, corrosion resistance, nitric acid corrosion, potentiodynamic polarization test, immersion test, mechanical alloying, X46Cr13 martensitic steel

## Abstract

This paper presents corrosion resistance results of a 12Cr ferritic ODS steel (Fe-12Cr-2W-0.5Zr-0.3Y_2_O_3_) fabricated via a powder metallurgy route as a prospective applicant for fuel cladding materials. In a spent nuclear fuel reprocessing facility, nitric acid serves as the primary solvent in the PUREX method. Therefore, fundamental immersion and electrochemical tests were conducted in various nitric acid solutions to evaluate corrosion degradation behavior. Additionally, polarization tests were also performed in 0.61 M of sodium chloride solutions (seawater-like atmosphere) as a more general, all-purpose procedure that produces valid comparisons for most metal alloys. For comparison, martensitic X46Cr13 steel was also examined under the same conditions. In general, the corrosion resistance of the 12Cr ODS steel was better than its martensitic counterpart despite a lower nominal chromium content. Potentiodynamic polarization plots exhibited a lower corrosion current and higher breakdown potentials in chloride solution in the case of the ODS steel. It was found that the corrosion rate during immersion tests was exceptionally high in diluted (0.1–3 M) boiling nitric acid media, followed by its sharp decrease in more concentrated solutions (>4 M). The results of the polarization plots also exhibited a shift toward more noble corrosion potential as the concentrations increased from 1 M to 4 M of HNO_3_. The results on corrosion resistance were supported by LSCM and SEM observations of surface topology and corrosion products.

## 1. Introduction

Nitric acid (*aqua fortis*, HNO_3_) is a strong and highly corrosive mineral acid widely utilized in various industrial processes. Concentrated HNO_3_ (~16 M) is also a powerful oxidizing agent, especially at elevated (>70 °C) temperatures, but its oxidizing properties are lost when it is diluted below approximately 2 M [1,2]. It is a crucial chemical in many industries due to its potent oxidizing properties and its ability to react with a diverse range of substances, forming highly soluble nitrate salts [1]. Despite its corrosive and potentially hazardous characteristics, the versatility of nitric acid is evident in its essential role across modern industry.

In facilities for reprocessing spent nuclear fuel, diverse nitric solutions are utilized at different stages of the well-established PUREX (Plutonium Uranium Redox EXtraction) process, which aims to separate fission byproducts by their dissolution in boiling nitric acid at various concentrations (1–14 M), followed by the extraction of the fuel for recycling [3,4,5]. Developed in the mid-20th century, PUREX remains the dominant method for separating uranium and plutonium from fission products in spent nuclear fuel and involves several key steps: (i) dissolution of spent nuclear fuel, (ii) solvent extraction, (iii) scrubbing and stripping, and (iv) final purification and conversion. This method is crucial for both reducing the volume and radiotoxicity of nuclear waste and recovering valuable fissile materials for reuse, thereby enhancing the sustainability of the nuclear fuel cycle by reducing the demand for freshly mined uranium [6,7]. Reprocessing activities involving spent nuclear fuel (SNF) produce radioactive waste liquors, which undergo concentration by evaporation before being stored for subsequent conversion into solid waste forms. These highly active waste liquors are nitric acid-based and are commonly processed and stored in stainless steel tanks. Due to the operation at elevated temperatures and the presence of concentrated nitric acid solutions containing oxidizing metal ions from spent fuel, the PUREX process is characterized by significant corrosiveness. Thus, there is an urgent need for equipment materials with exceptional resistance to nitric acid in reprocessing plants. The ultra-low carbon austenitic stainless steels, titanium alloys, and zirconium alloys are currently the preferred materials of choice used for this purpose in the world. However, stainless steel and zirconium alloys exhibit susceptibility to intergranular corrosion [4,8] and stress corrosion [9], respectively, which seriously limits their utilization.

Reduced activation ferritic oxide dispersion-strengthened (RAF ODS) steels are a group of promising structural materials, especially for applications involving elevated temperatures. Their enhanced properties are caused by a unique microstructure, which is characterized by a fine dispersion of oxide particles, acting as obstacles to migrating grain boundaries and dislocations. This pinning effect strengthens the metallic matrix and creep properties [10,11]. Despite fabrication challenges, the benefits of ODS steels in high-performance environments make them invaluable in power generation sectors. In particular, these alloys have emerged as potential materials for cladding tubes due to their favorable combination of high–temperature strength and resistance to irradiation. Resistance to radiation-induced swelling is attributed to their fine microstructure, which also serves as a sink for irradiation defects by trapping helium in fine-scale bubbles [12]. Following use, spent fuel pins are fragmented and subjected to immersion in a heated nitric acid solution to extract the fuel during reprocessing [13]. To assess the potential impact of corrosion products originating from cladding tubes on the reprocessing procedure, it is necessary to evaluate their corrosion behavior. For the ODS steels to be effectively utilized, their overall performance must meet certain criteria in the future. This includes enhanced corrosion resistance, particularly important for advanced nuclear energy systems where improved resistance to corrosion is crucial for cladding and core structural materials working in corrosive environments.

The corrosion behavior of ferritic stainless steels has been extensively studied, and it is generally agreed that oxide growth depends on many factors, mainly alloy composition, type of corrosive medium, temperature, and exposure time [14]. The impact of alloy composition on the oxidation characteristics of engineering alloys can be complex, especially when considering the influence of minor alloying and impurity elements [15]. In general, the steels’ enhanced resistance to corrosion is achieved through the addition of >12 wt.% chromium, facilitating the formation of a protective passive film, approximately 50 atomic layers thick, characteristic of stainless steels. This oxide film develops when the material is exposed to a corrosive medium, and it possesses self-repairing properties in typical environmental conditions, effectively shielding the steel from continued corrosion [13,16]. Ferritic stainless steels form chromium oxide, iron oxides, and/or their combinations, such as iron–chromium oxides. It was proposed that at the beginning of Fe–Cr alloy oxidation, iron oxide (Fe_2_O_3_) forms initially, subsequently converting with time to Fe-rich corundum oxide (Fe_2_O_3_–Cr_2_O_3_), and, finally, Cr-rich oxide [17].

Summarizing, the corrosion behavior of ODS steels in nitric acid environments is a critical concern, necessitating further detailed investigation. Hence, the present work was undertaken to explore and investigate the corrosion resistance behavior of 12Cr ferritic ODS steel in different electrolytic environments (HNO_3_ and NaCl solutions) by means of immersion and electrochemical tests. The results were compared to widely–utilized X46Cr13 martensitic steel, serving herein as a reference material, and studied under identical conditions.

## 2. Materials and Methods

### 2.1. Materials

#### 2.1.1. 12Cr RAF ODS Steel

A 12Cr RAF ODS alloy of the nominal chemical composition of Fe-12Cr-2W-0.5Zr-0.3Y_2_O_3_ was fabricated via the powder metallurgy route, which involved mechanical alloying (MA) of high purity, commercial metallic elemental powders, and nanometric Y_2_O_3_ (Thermo Fisher Scientific, Waltham, MA, USA) in a planetary ball mill Pulverisette 6 (Fritsch, Idar-Oberstein, Germany), and subsequent sintering by spark plasma sintering (SPS). During SPS, the consolidation of powders is influenced by a combination of both pressure and temperature, accompanied by the passage of an electric current [18]. The MA process required 40 h of milling (in the 10 min milling–20 min rest regime), which was the necessary time to obtain a solid solution, later confirmed by X-ray diffraction (XRD). The size of elemental as well as ball-milled powders, in terms of the De Brouckere mean diameter $d_{43}$, was controlled by using the laser particle sizer Fritsch ANALYSETTE 22 MicroTec plus. The measured mean size of powders, when MA was finished, was $d_{43}$ = 62.5(5) µm.

MA was conducted under a protective, high-purity (5N) Ar atmosphere (Air Products, Warsaw, Poland), using a 500 mL stainless steel vial and Ø 10 mm balls (~4 g each), preserving the ball-to-powder (BPR) mass ratio of 10:1 and rotational speed of ω = 300 1/min. Each batch produced around 50 g of a powder mixture. Whole ball milling equipment (planetary mill, vial, and balls) used in this study was manufactured by Fritsch. All operations involving powders subjected to MA were performed in an Ar-filled glovebox to minimize their contamination.

The SPS sintering was performed by pouring around 25 g of the ball-milled powders into a Ø 25 mm graphite dye, applying a pressure of 25 MPa, heating the sample to 1075 °C, and holding for 10 min. Obtained sinters were coarsely ground using a downdraft table to remove the graphite residue layer and then cut into smaller specimens (Ø 8 mm) using an electrical discharge machining (EDM) AU-300iA (AccuteX, Taichung, China).

#### 2.1.2. X46Cr13 Martensitic Stainless Steel

The experimental results obtained for ODS alloy were compared to the chromium martensitic stainless steel EN X46Cr13 grade (1.4034, AISI 420C) in an as-received, softened state (IK Stal, Jastrzębie-Zdrój, Poland), serving herein as a reference material. Unfortunately, the details explaining how the as-delivered state of material was achieved are unknown to the authors. This alloy was chosen due to several reasons. Most importantly, it has a similar Cr content to ODS steel and does not contain significant amounts of alloying additives besides Cr and C, which could affect its corrosion behavior. Also, it contains a notable C content, resulting in the occurrence of precipitates, although vastly different in morphology as compared to ODS steel. Generally, the carbon content of ~0.45% provides a compromise between high hardness and corrosion resistance for many applications [16,19]. Additionally, it is a well-known, basic alloy, inexpensive to produce and widely utilized in the industry, predominantly employed in a quenched and tempered state. It is extensively used across various engineering sectors, in particular, cutlery (kitchen and surgical blades), pipeline transport, valves, pumps, etc. [19]. This steel is commonly acknowledged for its high hardness (which can exceed 700 HV in a hardened state [20]) and decent corrosion resistance. Given its resilience, it is an apt choice for environments prone to high corrosion, such as those encountered in geothermal power plants with their corrosive thermal water.

The density of both 12Cr ODS and X46Cr13 alloys was measured on the basis of the Archimedes method, using high-precision XS205 analytical balance (Mettler Toledo, Columbus, OH, USA). The obtained results were 7.762(48) g/cm^3^ and 7.638(23) g/cm^3^ for the ODS and X46Cr13 alloys, respectively. Microhardness measurements were performed using a digital microhardness tester (Sinowon, Dongguan, China) under 25 g of load (HV0.025) and yielded 437(28) HV and 214(5) HV for 12Cr ODS and X46Cr13, respectively. Finally, the chemical composition of the materials was determined using a Thermo Fisher Scientific ARL QUANTRIS optical emission spectrometer and is presented in Table 1.

### 2.2. Immersion Test

Eleven solutions of varying HNO_3_ concentrations (0.1–14 M) were prepared in order to carry out the immersion test and to determine corrosion rates. The concentrations were chosen on the grounds of a model case of spent fuel leaching and reflect the different stages of spent fuel dissolution, from extensive to slight [13]. The corrosion rate (CR) by immersion test was calculated according to ASTM guidelines [21,22].

All specimens were Ø 8 mm cylinders with a total surface area of around 2 cm^2^. Shortly before the test, the samples were mechanically polished to eliminate the oxidized layer. The ratio of the solution volume to the samples’ surface area was around 12.5 cm^3^/cm^2^ in each test. Specimens were put into hot (~95–100 °C) solutions of HNO_3_ for 0.5 h, which was the necessary time to stabilize the process. Sample weight was evaluated prior ($m_{0}$) and after ($m_{f}$) the immersion, using an analytical balance, and the corresponding mass loss $m_{loss}$ was converted to corrosion rate.

### 2.3. Potentiodynamic Polarization Test

Electrochemical measurements consisting of pitting corrosion tests by dynamic polarization were carried out in a conventional electrochemical cell consisting of a 150 cm^3^ volume cell, open to the air, and a three-electrode system. A cylindrical Ø 8 mm working electrode (tested sample) was embedded in an isolating rubber sleeve so that only the desired surface was exposed to the solution (exposed area of 50.3 mm^2^). The counter electrode (XM140) was platinum foil with a total surface area of 128 mm^2^ (Radiometer Analytical, Villeurbanne, France) and a saturated calomel electrode (SCE, $E_{SCE}^{0}$ = +0.248 V vs. SHE at 20 °C) was used as a reference electrode, directly in contact with the working solutions. The potential of the working electrode was controlled by VoltaLab PGP201 potentiostat, and the response current was recorded in VoltaMaster 4 software (Radiometer Analytical). Measurements were performed using a scan rate of 3 mV/s. 

The temperature of the corrosion cell was controlled thermostatically using a water bath ICC basic (IKA–Werke, Staufen im Breisgau, Germany) and maintained at 20 °C during all the tests. The electrolytes were prepared with purified water of >18.2 MΩcm resistivity using Millipore Direct-Q water purification kit (Merck, Rahway, NJ, USA) and analytical grade sodium chloride and concentrated (65%, 14 M) nitric acid (Chempur, Piekary Śląskie, Poland). The tests were carried out with three different corrosive solutions: 0.61 M NaCl, 1 M HNO_3_, and 4 M HNO_3_. Experiments in NaCl were performed additionally as general, all-purpose tests that produce valid comparisons for most metals [23], as it corresponds to the mean salinity of seawater (~3.5 wt.% NaCl) and is commonly used in other studies. The total volume of the electrolyte in each experiment was 70 mL, and it was replaced with a fresh solution after each test. The tested samples were ground and polished using a set of emery papers (800–3000 grade) and 0.05 μm alumina slurry to achieve a mirror surface finish. Prior to the experiment, the samples were cleaned in an ultrasonic bath for 10 min in ethanol, washed with water, wiped, and immersed in the test solution shortly after. The three-electrode system was immersed in stagnant (non-stirred) solutions for 2 h before starting measurements in order to reach a steady-state value for the open-circuit potential (OCP).

**Figure 1:**
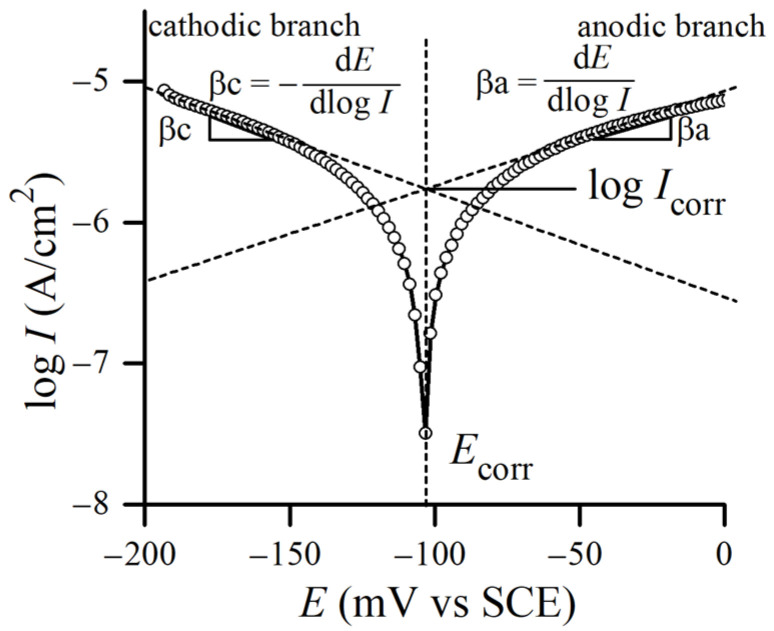
Tafel extrapolation with fundamental parameters used to calculate corrosion behavior (after [27,28]).

Corrosion current density $I_{corr}$ was obtained by the Tafel extrapolation method (Figure 1) [24], utilizing a well-known Stern–Geary equation [25,26]. As shown in Figure 1, the intersection of the straight lines on anodic and cathodic polarization curves corresponds to $I_{corr}$, whereas $E_{corr}$ is the corrosion potential. Polarization resistance $R_{p}$ was estimated as the slope of the polarization curve in a narrow potential region (±10 mV) around $E_{corr}$.

### 2.4. Microstructure and Phase Composition

The surface micro-morphology of selected samples before and after corrosion tests was observed microscopically by means of two imaging techniques, scanning electron microscopy (SEM) and laser scanning confocal microscopy (LSCM), using Thermo Fischer Scientific Phenom XL and Lext OLS 4000 (Olympus, Tokyo, Japan) microscopes, respectively. The observed surface and precipitates were analyzed by energy-dispersive X-ray spectrometry (EDS).

**Figure 2:**
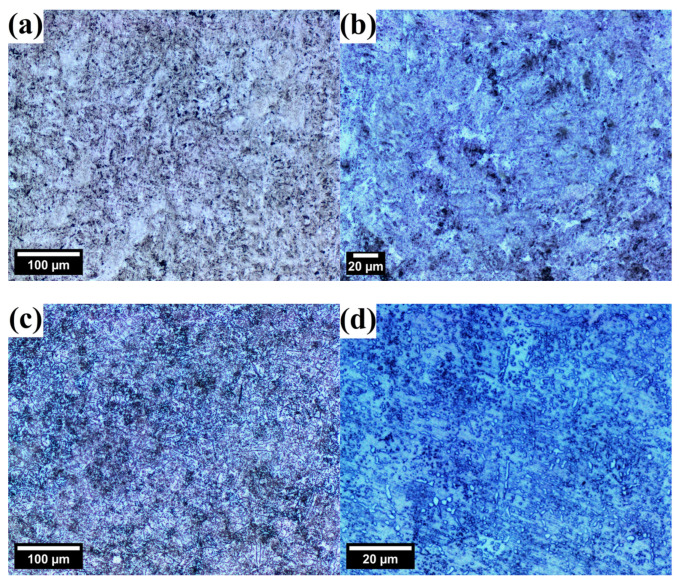
Optical micrographs presenting the uncorroded microstructures of 12Cr ODS (**a**,**b**) and X46Cr13 (**c**,**d**) steels (etched using waterless Kalling’s reagent).

The general, the optical microstructures of tested steels prior to corrosion tests are depicted in Figure 2. ODS steel is characterized by a very fine, ferritic microstructure with visible second-phase particles, presumably originating from mill contamination during the MA (Figure 2a,b). The microstructure of X46Cr13 steel is also ferritic, decorated with uniformly dispersed, oval pearlites around 1–5 μm in diameter (Figure 2c,d).

The XRD data was collected to evaluate the samples’ phase composition. A laboratory D8 Advance powder diffractometer was used (Bruker, Karlsruhe, Germany), working in Bragg–Brentano focusing geometry, equipped with a Cu radiation source produced at 40 kV/25 mA. Each powder pattern was gathered in the 25°–125° 2θ range using a 5 s acquisition time per 0.01° step size. The obtained peak profiles were processed by the Whole Powder Pattern Modelling (WPPM) method to extract basic microstructural data, particularly in terms of a lattice constant ($a_{0}$) and a crystallite size (d) [10,29].

**Figure 3:**
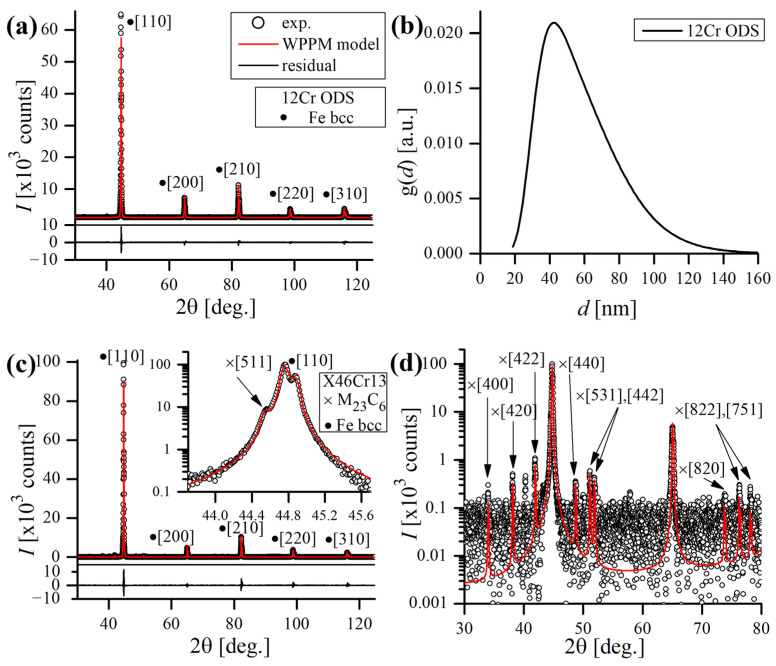
Experimental (scatter) and modeled (red line) XRD results of the ODS (**a**) and X46Cr13 (**c**,**d**) steels, lognormal distribution of the domain size of the ODS steel (**b**) and highlighted minor reflections of the carbide phase detected in the X46Cr13 steel (**d**). Residual (black line) at the bottom shows the disagreement between the model and experimental data.

Experimental and modeled XRD data of both the studied alloys is presented in Figure 3. The lattice constants of $a_{0}$ = 2.874011(8) Å and 2.873325(4) Å were determined for the 12Cr ODS and X46Cr13 steels, respectively, which are typical values for ferritic bcc alloys ($a_{0}$ ≈ 2.8665 Å for a pure Fe–bcc). The mean crystallite size (modeled by the lognormal distribution $g\left(d\right)$ of spherical crystalline domains, Figure 3b) of the ODS steel appeared to be d = 58 nm, which indicates that the ODS alloy retained a fine microstructure after sintering by the SPS. This is reasonable, considering the long-term ball milling and a brief sintering time, limiting the heat-induced grain growth and microstructure’s coarsening. On the other hand, the estimated mean domain size (by Dirac delta distribution) of the X46Cr13 steel was 391 nm, revealing a relatively coarse microstructure characteristic of the soft-annealed state. No clear strain-related broadening of the peak profiles was observed (i.e., the dislocation density ρ was <$10^{15}$/m^2^, below the threshold limit of XRD detection). In general, the microstructure of the ODS steel was found to be purely bcc, proving a proper formation of the single-phase solid solution during MA. In the case of the X46Cr13 steel, some minor peaks, originating from a carbide phase, were detected beside main ferritic bcc reflections (Figure 3c,d). The measured lattice constant of this phase was found to be $a_{0}$ = 10.605700(7) Å, corresponding to a M_23_C_6_-type carbide. As the lattice constant of Cr_23_C_6_ is 10.650(2) Å [30], the discrepancy between those values suggests a different or more complex chemical composition of the carbide inclusions, preferably of the Cr_23-x_Fe_x_C_6_ type, crystallizing in the fcc lattice [31]. 

## 3. Results

### 3.1. Immersion Test in Boiling 0.1–14 M HNO_3_

**Figure 4:**
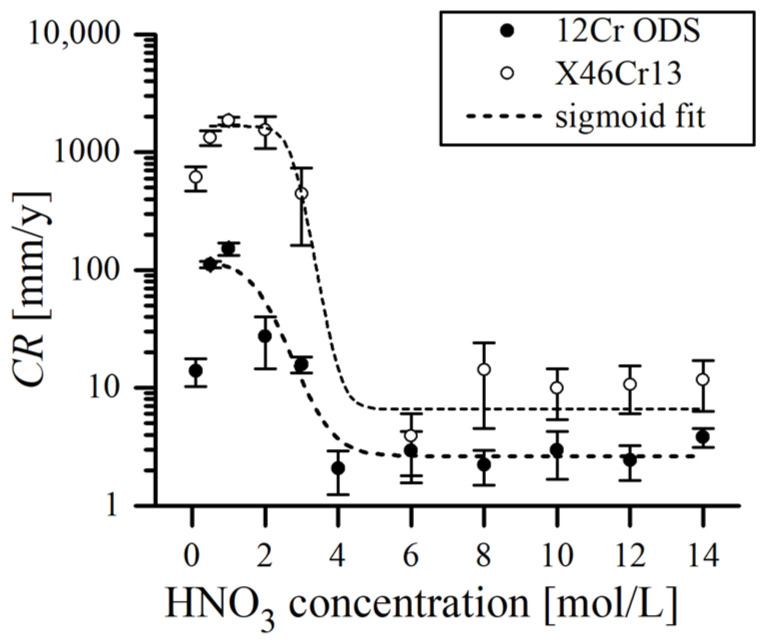
Dependence of corrosion rate of immersion test in boiling HNO_3_ solutions obtained for 12Cr ODS and X46Cr13 alloys.

From the results, it is clear that the corrosion rate CR in hot nitric acid solutions was strongly dependent on the molar concentration of acid. As a general rule, CR decreased as the concentration of HNO_3_ increased in the solution, which was also observed in other studies [13]. According to Figure 4, this tendency can be more precisely divided into four specific regions. Very diluted HNO_3_ (0.1 M, the weakest solution used in this study) still caused considerable degradation of the studied materials, as the measured CR was relatively high (13.9(3.7) and 612.4 (142.7) mm/y for ODS and X46Cr13, respectively). The region of 0.5–1 M is where both alloys exhibited the most aggressive acid attack, witnessed by the highest mass loss (CR = 151.8(19.1) and 1853.4(137.6) mm/y for ODS and X46Cr13, respectively), at 1 M HNO_3_. Solutions of 2–4 M constitute the transition region, where the sharp decrease in CR was observed, which then evolved into the plateau region as the acid concentrations further increased. Above 4 M of HNO_3_, the decrease in corrosion rate stopped and stabilized at ~2 mm/y and ~10 mm/y levels for ODS and X46Cr13, respectively, with very little variations around these values. Therefore, the CR data points plotted for the immersion test follow a characteristic S-shaped sigmoid curve (Figure 4).

In Figure 4, it can be noticed that the data points plotted for both alloys mimic the trend of each other. However, the reported CR values are roughly an order of a magnitude higher in the case of X46Cr13 martensitic steel, albeit it had around a 1 wt.% higher Cr content (Table 1). This can be explained by the addition of ~2 wt.% W to the ODS alloy, as it is suggested that W, similarly to Mo, could aid in passivation [13,32]. Also, some authors suggested that the presence of Y_2_O_3_ nanoclusters may have a role in suppressing corrosion in ODS steels by its beneficial effect on refining the grain size and hindering the growth of grains [33]. This is, however, doubtful, as Y_2_O_3_ dissolves in HNO_3_ and probably does not have significant benefit [34]. Some studies even report that the presence of fine dispersoids can be detrimental to the corrosion resistance in NaCl solutions and enhance pit nucleation as they occur in the passive film in discontinuities with less protection ability when compared to the continuous passive film in common ferritic steels [35]. 

Another explanation can be the possible discrepancy of the measured and “available” Cr content in X46Cr13 due to the affinity of Cr to form carbides. This is dependent on the applied heat treatment, strongly influencing hardness and corrosion resistance [16]. The optimal corrosion resistance of such alloys is attained through solution annealing, during which chromium carbides are completely dissolved [36,37,38]. Failure to achieve complete dissolution results in residual Cr carbides and, in consequence, Cr depletion, thereby lowering its overall concentration in the solid solution [20]. The occurrence of Cr-depleted zones in these alloys further deteriorates corrosion resistance [39]. Therefore, the presence of a high proportion of Cr carbides, commonly observed in the soft-annealed state of martensitic stainless steels, which is the case in this study, is unfavorable and leads to reduced corrosion resistance. On the contrary, when the steel is properly hardened, all carbon is incorporated into the martensite’s solid solution, maximizing corrosion resistance. This effect is more pronounced in steels with a higher carbon content or lower chromium content, owing to a greater fraction of chromium carbides in the structure [36,37].

**Figure 5:**
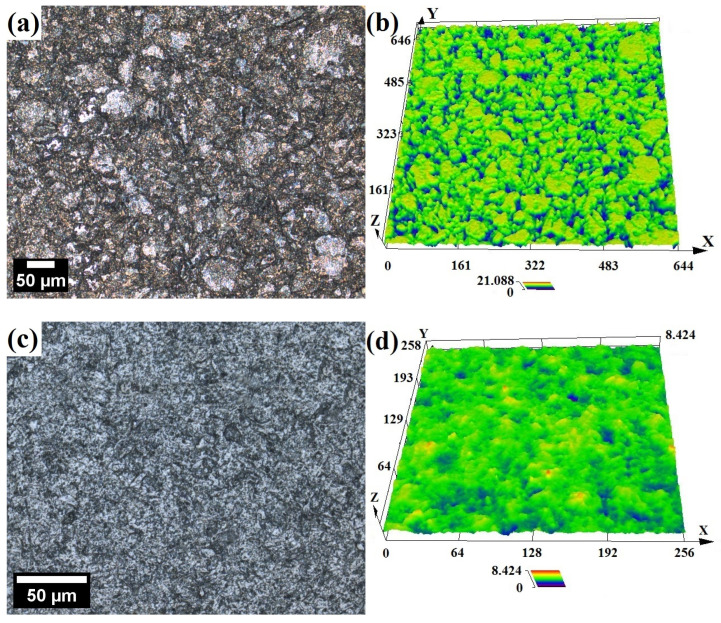
Laser confocal micrographs and surface topology of 12Cr ODS (**a**,**b**) and X46Cr13 (**c**,**d**) samples after immersion in boiling 1 M HNO_3_ solution.

The optical micrographs with corresponding surface topology maps of the samples after the immersion test in 1 M of HNO_3_ are presented in Figure 5. In addition, standardized surface roughness parameters of corroded samples, in particular the average roughness ($R_{a}$) and maximum peak-to-valley height of the profile ($R_{z}$), were evaluated by line scans (six for each sample, over a length of about 200 μm) on LSCM topology maps and are presented in Table 2.

From the topology maps, it can be concluded that, despite a much lower corrosion rate, the surface of the ODS steel is rougher, porous and has a groove-like network structure with deeper valleys, as compared to X46Cr13 (Figure 5). It is also evidenced by much higher $R_{a}$, $R_{z}$ values obtained for ODS steel immersed in 1 M of solution (Table 2). This suggests that in the case of X46Cr13, the surface dissolution is more uniform, whereas it is more localized in ODS steel. In particular, ODS surface topology resembles microstructure exposing prior particle boundaries (PPBs), which are often extensively decorated with Cr-rich precipitates [40], thereby lowering the Cr content in these areas and making it susceptible to acid attack. PPBs are harmful structures because they weaken the metallurgical bonding between powder particles [41]. Also, the intergranular region is where residual porosity is present in materials processed via powder metallurgy, further deteriorating corrosion resistance.

**Figure 6:**
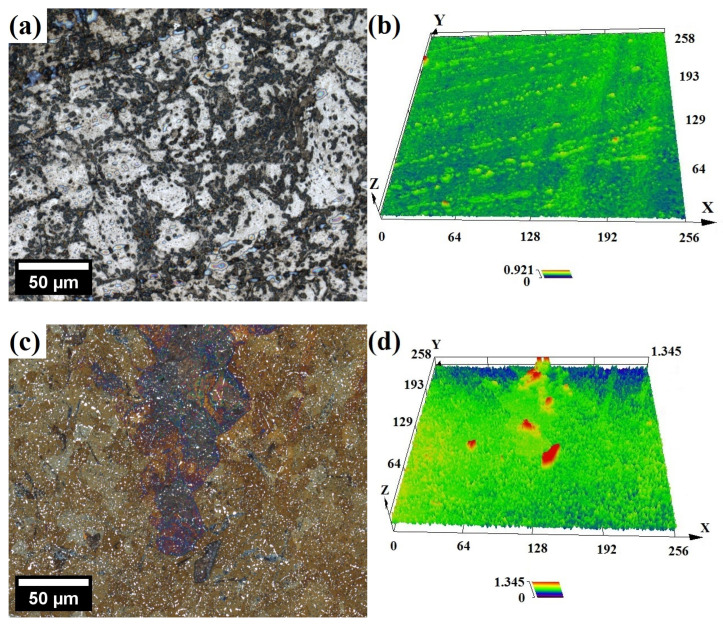
Laser confocal micrographs and surface topology of 12Cr ODS (**a**,**b**) and X46Cr13 (**c**,**d**) samples after immersion in boiling 14 M HNO_3_ solution.

Similar micrographs after the immersion test in 14 M of HNO_3_ are presented in Figure 6. It is obvious that the acid corrosive effect on the surface is much less pronounced, witnessed by very shallow valleys, which is consistent with the much lower corrosion rate in concentrated HNO_3_ as compared to the diluted solutions (Figure 4, Table 2). As per cited sources, the rate of corrosion is relative to the dissolution of Fe ions until a Cr-rich, protective layer fully coats the surface and slows down the exchanges between the bulk metal and the solution [13,42]. In other words, after immersion, a certain amount of Fe reacts rapidly with HNO_3_ before the surface becomes passivated, and the reaction is inhibited. The higher the Cr content is, the more resistant the ODS steel is to HNO_3_ corrosion [42]. A more concentrated HNO_3_ solution has the capability to create a thin Cr passive film with a higher concentration of Cr within [34,43]. Therefore, the surface of the specimens subjected to concentrated HNO_3_, owing to its strong oxidizing properties, could be passivated faster with little dissolution. On the other hand, the lower conductivity and limited oxidizing properties of the diluted acid are not sufficient to induce passivity [44]. Above 8 M, an autocatalytic reduction of HNO_3_ into strongly oxidant species such as NO_2_ occurs, promoting rapid surface oxidation and transition to the passive corrosion domain, thus limiting the dissolution rate [33,45,46]:

(1) $$4{HNO}_{3}\to 4{NO}_{2}+{2H}_{2}O+O_{2}$$

However, it should be underlined that the immersion tests conducted in this study were relatively short (30 min). In longer tests (several hours at least), more oxidizing conditions prevailing in the more concentrated HNO_3_ medium may result in decreased passive film stability and, consequently, accelerate the dissolution rate [42,46,47]. It was found that during long exposure times in HNO_3_, the chromium protective layer is slowly depleted from the surface according to the following reaction [46]:

(2) $${Cr}_{2}O_{3}+6{HNO}_{3}+{9H}_{2}O\to {{2Cr(NO}_{3})}_{3}+{12H}_{2}O$$

According to other studies, the addition of Al is beneficial to the corrosion resistance of ODS alloys as Al_2_O_3_ has greater stability than Cr_2_O_3_. Hence, the resistance to dissolution in acid media is improved when the passive film is enriched with Al_2_O_3_ [46,47]. 

According to Hultquist et al. [48], the dissolution rate of pure Fe in 9.2 M of HNO_3_ is around two orders of magnitude higher as compared to a 1 M solution, whereas the dissolution rate of pure Cr is only barely affected by the acid concentration. In 9.2 M of HNO_3_, the CR of Fe is more than $10^{6}$ times higher compared to Cr. Therefore, when considering the low dissolution rate of pure Cr and the fact that the dissolution rate of pure Fe is vastly greater, it is reasonable to expect a rapid accumulation of Cr on the surface of stainless steel upon exposure to the concentrated HNO_3_ solution. 

In the case of the ODS alloy (Figure 6a), the surface is mostly silver, which suggests that it was passivated almost immediately. In opposition, the surface of martensitic steel is tinted brown (Figure 6c), which indicates that it could not have been passivated instantly. The visible brown film observed in microscopic images likely represents a Fe-rich oxide layer. It was formed during immersion when Fe preferentially dissolved from the surface prior to the formation of a Cr-rich passive layer. Pure Fe also has the ability to passivate in concentrated HNO_3_ due to the formation of the ferric acid film (or a related higher valence Fe compound). This film is, however, fragile and breaks down by cathodic reduction, mechanical shock, or the presence of halide ions [44]. Fe-rich oxide layer might not offer complete protection for the metal surface; however, its growing thickness can impede ion migration and/or diffusion between the metal surface and the solution, decreasing the corrosion rate. Ultimately, Cr accumulates sufficiently on the metal surface as a result of the preferential dissolution of Fe, leading to the passivation of the metal surface. 

In the study covering the 9Cr, 14Cr, and 18Cr ODS steel compositions [42], it was found that Cr and Fe are present on the surface of uncorroded samples in both metallic and oxidized states. A thin (below 10 nm), native (due to oxidation by the ambient air) oxide layer covers the alloy’s surface, which is composed of Cr_2_O_3_, Fe_2_O_3_, and FeO(OH) and has a similar composition (in at.%) to steel, with only a little higher Cr content than that corresponding to its bulk alloy concentration. However, after the corrosion tests in HNO_3_ (9 M, 90 °C), the thickness of the oxide layer rises (exceeds 15 nm), and the Cr content on the surface becomes vastly larger (around 90% of Cr relative content). It was noticed that the more the steel contains Cr, the richer in Cr is the oxide layer, which explains why steel containing more Cr is more effectively protected. In another study, it was reported that the thickness of an oxide film of Fe-26Cr and Fe-26Cr-2Mo ferritic steels immersed in HNO_3_ is in the range of 0.8–1.0 nm and rich in Cr (~80% of relative content, as measured by electron spectroscopy) [48]. Also, the chemical composition of the Cr-rich oxide layer was found not to be affected either by the HNO_3_ concentration or by the temperature [42]. Other studies also report Cr enrichment and Fe depletion in the oxide layer, caused by selective dissolution and high mobility of Fe. This results in chromium oxide being the primary oxide species in the passive film after corrosion tests in HNO_3_ [34,47]. 

**Figure 7:**
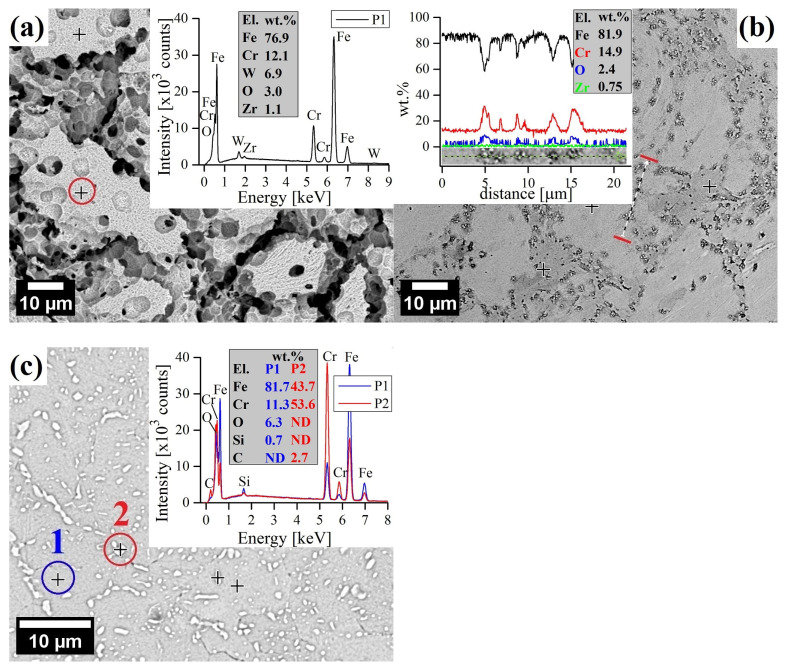
SEM surface micrographs after immersion test in boiling HNO_3_ solutions: 12Cr ODS (1 M) with point EDS analysis (**a**), 12Cr ODS (14 M) with line scan EDS (**b**), X46Cr13 (14 M) with point EDS analysis (**c**). The presented analysis spectra were obtained in the color–marked points/lines.

SEM observations of corroded surfaces were carried out to study the morphology of corrosion products. After immersion in 1 M of HNO_3_, the surface of the ODS alloy exhibited a near complete removal of the surface structures, exposing a large granular structure with an elevated level of O without any visible precipitates (Figure 7a). It is evidence of significant dissolution of the surface, corresponding to the high corrosion rate (Figure 4). When dissolution serves as the primary corrosion mechanism, the numerous grain boundary channels present in the fine-grained structure of 12Cr ODS steel will expedite the dissolution of alloy elements [49]. The energy of the grain boundary is high, and therefore, it easily engages in chemical reactions [50]. Corrosion exists primarily within the grain boundaries because they are the regions where defects like dislocations, distortions, segregation, etc., are accumulated, making them a preferred site for the onset of corrosion [51]. It was found that an excessively high density of sub-grain boundaries, typical for ODS alloys obtained via powder metallurgy, accelerates corrosion [52]. Therefore, it is evident that corrosion is intensified along the grain boundary area, and thus, grain boundary corrosion appears to be the primary method of chemical attack (Figure 7a). As demonstrated earlier by LSCM, the surface is severely roughened, with clearly visible, round cavities of a few µm, which may result from local differences in chemical composition and be the local areas of selective acid attack. 

In contrast, the surface of 12Cr ODS steel after the test in 14 M of HNO_3_ also shows well-defined etch along grain boundaries but without clearly visible roughness and extensive material dissolution (Figure 7b). What is more, EDS analysis confirmed the presence of Cr-rich oxides, indicating occurrence of the surface passivation. Similarly, elevated levels of O were found on the surface of X46Cr13 after immersion in 14 M of HNO_3_, with extensive decoration of white carbide precipitations rich in Fe, Cr, and C (Figure 7c). 

### 3.2. Potentiodynamic Polarization Test in 1 M and 4 M HNO_3_

**Figure 8:**
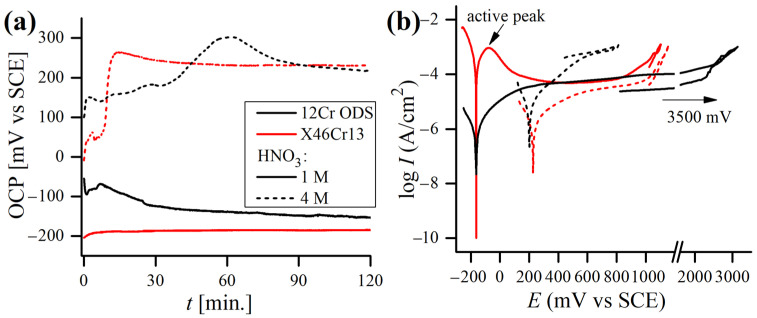
Typical open circuit potential versus time (**a**) and potentiodynamic polarization curves (**b**) in 1 M and 4 M HNO_3_ concentrations obtained for 12Cr ODS and X46Cr13 alloys.

The measured OCP and potentiodynamic polarization curves of 12Cr and X46Cr13 alloys obtained at different concentrations of 1 M and 4 M of HNO_3_ at 20 °C are shown in Figure 8. The concentrations of HNO_3_ solutions in the polarization test were chosen on the basis of the immersion test results (Figure 4) and represent the most corrosive HNO_3_ solution (1 M), while 4 M was the lowest concentration in which the passivation of materials occurred, and the corrosion rate dropped significantly. In Figure 8a, the effects of HNO_3_ concentrations on both alloys show active potential in 1 M of solution, but in 4M of solution, the OCP is shifted towards more noble values, which is caused by a change in anodic/cathodic reaction rates and usually is a sign of high passivating ability. Generally, the increase in $E_{corr}$ can be attributed to either an increase in the cathodic process or inhibition of the anodic one [53]. Detailed information on the reaction kinetics can be derived from the polarization curves (Figure 8b) using anodic/cathodic Tafel slopes ($\beta_{a}$, $\beta_{c}$, Table 3). The higher the $\beta_{a}$ and $\beta_{c}$ values (resulting in less steep slopes when the potential is plotted on the abscissa), the higher the activation energy required for the reaction to occur, and the slower the reaction rate is. In the case of both alloys, a stronger HNO_3_ solution caused the reduction of $\beta_{a}$, suggesting an acceleration of the anodic process, caused by a more oxidizing nature of the solution. Also, the anodic dissolution (reflected in the value of $I_{corr}$ [54]) increased with the increase in the acid concentration in the case of the ODS steel. Hence, this shift is probably a result of an increase in the cathodic process, and so $E_{corr}$ rises when acid concentration is higher [42]. 

The measured OCP stabilizes almost immediately upon immersion in 1 M of acid and does not change significantly during the test, which can be attributed to the rapid formation of a stable oxide film [34]. However, in a 4 M medium, OCP requires more time to stabilize, which may signal the occurrence of more complex reactions in the electrochemical cell caused by different balances of autocatalytic reduction mechanisms involved in HNO_3_. HNO_3_ is reduced by a chemical reaction that regenerates the electroactive compounds:

(3) $${HNO}_{3}+NO↔{HNO}_{2}+{NO}_{2}$$

The more concentrated the acid is, the faster reaction (1) occurs, enhanced by the autocatalytic mechanism [13]. For lower concentrations (<6 M), the reduction of HNO_2_ is the main reaction [34,55]:

(4) $${2HNO}_{2}↔{NO}_{2}+NO+H_{2}O$$

The NO_2_ oxidant generated in the reaction (2) is subsequently adsorbed onto the metal surface, where it gains an electron from the metal dissolution to form the ${NO}_{2}^{-}$ anion [34,56].

(5) $${NO}_{2}+e^{-}\to {NO}_{2}^{-}$$

Further, a passive behavior (i.e., the regions where the oxidation current is nearly independent of the potential) can be noticed on both alloys’ polarization curves obtained in the 1 M of HNO_3_. Both alloys become passivated at potential values exceeding 400 mV/SCE. However, the breakdown potential of ODS steel is much higher (~1800 mV/SCE) than of the martensitic one (~900 mV/SCE). In the 1 M solution, both alloys exhibit very similar $E_{corr}$ values, although the martensitic steel has a very low polarization resistance $R_{p}$ and, as a consequence, a very high corrosion current density $I_{corr}$, more than 250 times higher than the ODS steel (Table 3). On the other hand, the current stable region is not observed on the polarization curves obtained in the 4 M acid in the case of the ODS steel but can be distinguished for the martensitic steel, which becomes transpassive at around 1000 mV/SCE (Figure 8b). It is worth underlining that the repassivation potential for both tested alloys in the HNO_3_ solutions was not observed.

Although the corrosion resistance of martensitic steel during the polarization test in 1 M of HNO_3_ was vastly worse as compared to 12 Cr ODS steel, it was not the case in the 4 M test solution, as both alloys performed similarly (Table 3). When the concentration of acid was increased to 4 M, the CR of X46Cr13 steel dropped by around two orders of magnitude, whereas the CR of 12Cr ODS slightly increased. With the increase in the HNO_3_ concentration, a higher OCP and $E_{corr}$ may lead to a more oxidizing nature, and by that lead to a less protective nature of the passive film and escalate anodic dissolution [34].

### 3.3. Potentiodynamic Polarization Test in 0.61 M NaCl

**Figure 9:**
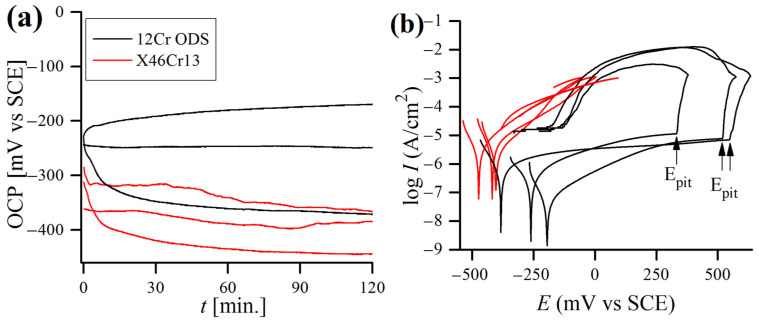
Typical open circuit potential versus time (**a**) and potentiodynamic polarization curves (**b**) in 0.61 M NaCl solution, obtained for 12Cr ODS and X46Cr13 alloys at 20 °C.

The typical OCP and potentiodynamic polarization curves of both steels are plotted in Figure 9, with corresponding mean values (average of at least 5 samples) of the polarization data being reported in Table 4. Both alloys exhibited a stable OCP (Figure 9a) from the moment of immersion up to the end of the test. It is clear that $E_{corr}$ was more noble for the ODS alloy. The large anodic peak observed for both steels can be assigned to the oxidation of Cr to the thermodynamically more stable Cr^3+^ [57]. 

According to the results, the 12Cr ODS steel had almost ten times higher $R_{p}$ than the martensitic one, and, as a consequence, the calculated $I_{corr}$ was significantly lower for the 12Cr ODS steel (Table 4). None of the X46Cr13 samples demonstrated a clear passivity region. Nevertheless, in the case of 12Cr ODS steel, a passive region can be distinctly observed in all of the curves, indicating typical and similar passivation behaviors. The flat shape of the curves running in the passive area revealed that the oxide layer in the ODS steel was more protective than in the martensitic one, where a continuous oxidation of chromium took place. When the corrosion potential reached the values of ~300–500 mV/SCE, a fast increase in the current density was observed (Figure 9b). This rapid increase in current density is caused by the initiation of pitting corrosion and is characteristic of transpassive oxidation of chromium from Cr^3+^ to CrO_4_^2−^ [58]. Also, no repassivation potential was detected for any of the samples tested in the NaCl solution. The calculated mean $I_{corr}$ value for the ODS steel was similar to those obtained by other authors for similar ODS alloys tested in 0.61 M NaCl (collated in [59]). 

**Figure 10:**
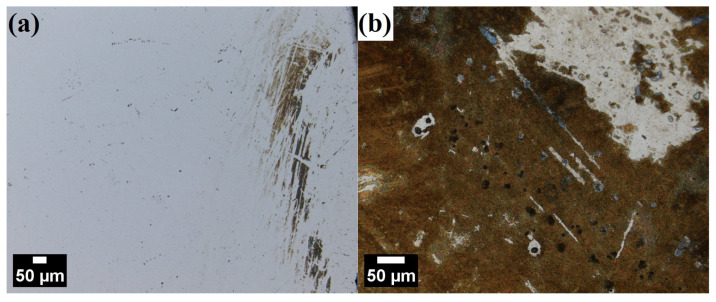
LSCM micrographs comparing general surface condition of 12Cr ODS (**a**) and X46Cr13 (**b**) steels after polarization test in 0.61 M NaCl.

General LSCM observations after polarization tests in NaCl revealed that the surface of ODS steel was largely unaffected, with only scarce occurrence of regions with visible corrosion attacks (Figure 10a). In contrast, the surface of martensitic steel was macroscopically affected and brown-tinted, with obviously visible corrosion products covering almost the entire sample (Figure 10b). 

**Figure 11:**
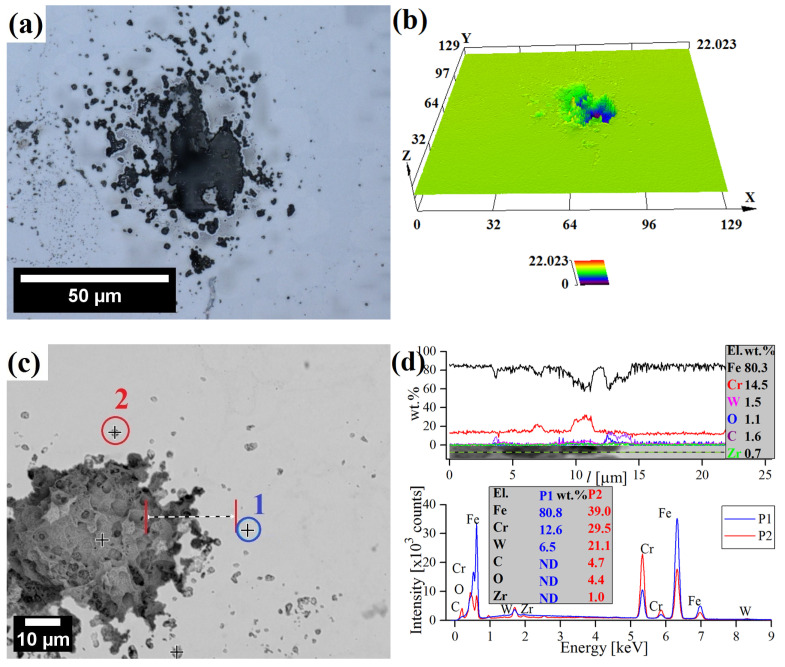
LSCM (**a**,**b**) and SEM micrographs (**c**,**d**) of surface corrosion with corresponding EDS analysis of 12Cr ODS steel after polarization tests in 0.61 M NaCl. The presented analysis spectra were obtained in the color–marked points/lines.

More in-depth observations revealed the presence of a few isolated corrosion pits on the surface of the ODS steel (Figure 11). Overall, the surface of ODS steel was mostly uninfluenced and featureless, with singular corrosion cavities around 40 μm wide and 20 μm deep, presumably in the regions where the Cr concentration was especially low or where residual porosity pre-existed and the corrosion was enhanced (Figure 11a,b). The main causes of Cr depletion sites, where pitting is initiated, are probably microstructural inhomogeneities and inclusions. However, the Cr-depleted zone around the inclusions cannot be the only factor responsible for the preferential pit initiation in their vicinity [60]. It is widely understood that the presence of aggressive anions detriments the passive film stability, causing its local breakdown, mostly at sites of local heterogeneities, and, as a consequence, pitting. The main factors, which are often quoted as responsible for the harmful effect of Cl^−^ anions are (i) the adsorption of Cl^−^ anions onto the passive film, resulting in its breakdown and the chemical dissolution of the oxide layer, and (ii) the penetration of anions in the film leading to weakening of the oxide bonds [61,62]. Point EDS analysis of precipitates near the corrosion pit shows that they are rich in Fe, Cr, W, C, and O in reference to the matrix (Figure 11c,d). The chemical composition of the corrosion pit did not deviate significantly from the matrix (Figure 11d).

**Figure 12:**
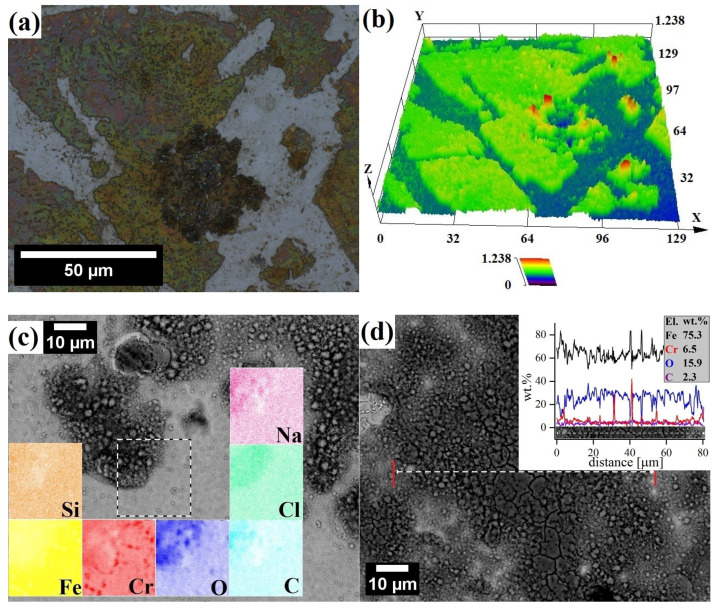
LSCM (**a**,**b**) and SEM micrographs (**c**,**d**) of surface corrosion with corresponding EDS analysis of X46Cr13 steel after polarization tests in 0.61 M NaCl.

There was an absence of significant corrosion pits in the X46Cr13 alloy; however, surface topology confirmed the presence of a macroscopic 0.6 µm-thick film on the martensitic steel (Figure 12), covering most of the surface. The SEM-EDS analysis confirmed that this film was cracked and porous, rich in Fe and O but poor in Cr (Figure 12c,d). It has been reported that 9-12Cr martensitic steels form a duplex oxide scale composed of a porous hematite/magnetite (Fe_2_O_3_/Fe_3_O_4_) layer [63]. Hematite tends to stick better to the surface than magnetite does, especially when there are obstacles like gaps or pores that limit the movement of iron during corrosion. When this happens, hematite forms on the outer layer of magnetite. The magnetite layer has columnar grains, while the hematite layer has smaller equiaxed grains. The outer layer of hematite may flake off due to compressive stress from oxide growth, while magnetite typically cracks because of the tensile stress from oxide growth (Figure 12d) [64].

## 4. Conclusions

In this study, the corrosion resistance, passive film compositions, and surface morphology of ferritic 12Cr ODS and X46Cr13 steels in different nitric acid and sodium chloride media were evaluated by means of immersion and electrochemical tests. During a short-term (30 min) immersion test in boiling HNO_3_ solutions, the corrosion rate was remarkably high in diluted (0.1–3 M) HNO_3_ solutions and decreased steeply in more concentrated (>4 M) acid, according to the sigmoid curve. In general, the corrosion rate measured in immersion tests was at least one to two orders of magnitude lower for the 12Cr ODS than for X46Cr13 steel, regardless of HNO_3_ concentration. During polarization tests in 1 M and 4 M of HNO_3_, the shift of open circuit potential towards more noble values was observed for both alloys. 

Polarization tests carried out in 0.61 M NaCl showed a slightly more noble corrosion potential and lower corrosion current density of the 12Cr ODS steel. Moreover, the surface morphology observations of the X46Cr13 steel revealed a massive cracked oxide layer composed mainly of ferrous oxides, which was almost non-existent in the ODS steel. However, in the ODS steel, singular, deeper corrosion pits were detected, signalizing a localized corrosion attack. The better corrosion resistance of the ODS steel, despite its lower nominal Cr content, can be attributed to the unfavorable, soft–annealed state of the X46Cr13 steel, with the occurrence of a high proportion of Cr-rich carbides, depleting the Cr content in the matrix and leading to reduced corrosion resistance.

## Figures and Tables

**Table 1:** Chemical composition of tested alloys determined by optical emission spectrometry. Values are in wt.%.

Alloy	Fe	Cr	W	Zr	C	Si	Mn	Ni
12Cr ODS	bal.	12.58	1.49	0.57	0.11	0.02	0.10	-
X46Cr13	bal.	13.53	-	0.01	0.46	0.29	0.54	0.19

**Table 2:** One-dimensional roughness parameters of samples after immersion tests in HNO_3_, evaluated by the LSCM observations. $R_{a}$ is the average roughness, $R_{z}$ is the maximum peak-to-valley height of the profile, and σ is the standard deviation, respectively.

Material HNO_3_ Solution	12Cr ODS 1 M	12Cr ODS 14 M	X46Cr13 1 M	X46Cr13 14 M
$R_{a}$ [μm]	1.483	0.045	0.167	0.109
σ [μm]	0.099	0.003	0.008	0.025
$R_{z}$ [μm]	10.698	0.427	1.222	0.757
σ [μm]	0.798	0.035	0.120	0.131

**Table 3:** Mean values of electrochemical parameters calculated from the polarization tests conducted in HNO_3_ solutions. Values in brackets correspond to standard deviation of measurements.

Material	$E_{corr}$ [mV/SCE]	$R_{p}$ [kΩcm^2^]	$\beta_{a}$ [mV/dec]	$\beta_{c}$ [mV/dec]	$I_{corr}$ [µA/cm^2^]
	1 M HNO_3_, 20 °C				
12Cr ODS	−133.0 (42.1)	17.9 (6.4)	125.7 (28.7)	122.5 (20.3)	1.7 (0.9)
X46Cr13	−171.5 (11.2)	0.055 (0.0006)	226.6 (135.8)	72.6 (21.7)	429.8 (159.1)
	4 M HNO_3_, 20 °C				
12Cr ODS	201.6 (27.2)	3.2 (1.1)	101.7 (18.4)	86.3 (13.5)	6.28 (2.22)
X46Cr13	227.2 (14.9)	15.7 (4.6)	175.7 (41.3)	118.8 (19.6)	1.96 (1.13)

**Table 4:** Electrochemical parameters calculated from the polarization tests conducted in NaCl. Values in brackets correspond to standard deviation of measurements.

Material	$E_{corr}$ [mV/SCE]	$R_{p}$ [kΩcm^2^]	$\beta_{a}$ [mV/dec]	$\beta_{c}$ [mV/dec]	$I_{corr}$
	0.61 M NaCl, 20 °C				
12Cr ODS	−392.5 (142.9)	43.8 (24.4)	94.3 (26.9)	81.0 (10.0)	0.68 (0.48)
X46Cr13	−450.2 (36.2)	5.0 (1.9)	73.4 (9.2)	50.0 (8.3)	2.89 (1.11)

## Data Availability

The data presented in this study are available on request from the corresponding author (K.N.). The data are not publicly available due to privacy.
